# Comparing efficacy and adherence of smartphone-guided exercises to conventional self-directed exercises for neck pain in office workers: A randomized controlled trial protocol

**DOI:** 10.1371/journal.pone.0329863

**Published:** 2025-09-19

**Authors:** Sana Salah, Anis Jellad, Manel Mili, Amr Chaabeni, Mohamed Hédi Bedoui, Helmi Ben Saad

**Affiliations:** 1 Department of Physical Medicine and Rehabilitation, Faculty of Medicine, Fattouma Bourguiba University Hospital, University of Monastir, Monastir, Tunisia; 2 Faculty of Medicine, Laboratoire Technologies et Imagerie Médicale LabTIM LR12ES06, University of Monastir, Monastir, Tunisia; 3 Faculty of Medicine of Sousse, University of Sousse, Farhat HACHED Hospital, Heart Failure (LR12SP09) Research Laboratory, Sousse, Tunisia; The Hong Kong Polytechnic University, HONG KONG

## Abstract

**Background:**

Self-exercises focusing on strength and endurance training, as well as self-mobilization, are effective in neck pain (NP). This study aims to investigate the differences in self-management using two workplace-based interventions: a smartphone application consisting of personalized neck exercises compared to conventional approach (self-exercises program on paper) in chronic NP office workers.

**Methods:**

The project is a prospective, superiority, randomized controlled trial. Fifty participants with chronic NP will be randomly assigned into the interventional group (IG, n = 25), utilizing the smartphone application, or the control group (CG, n = 25). The CG includes the use of a paper sheet with exercises and recommendations, and the IG includes the use of a smartphone application, which provides individualized exercise programs. Both protocols will last three months and will be preceded by an educational session at baseline for all participants. The main outcome measure comprises pain intensity evaluated according to the pain intensity number rating scale. Secondary outcomes are function evaluated according to the neck disability index, quality of life according to the short form 12, and participants’ adherence to self-exercises. Outcome measures will be collected at baseline, and one and three months of follow-up.

**Discussion:**

The current project will evaluate the effectiveness of a smartphone application consisting of personalized neck exercises, when compared with a conventional approach for self-rehabilitation. The smartphone application will allow to monitor the participants’ status and to help resolving the problem of adherence to self-exercises in chronic NP. Despite some limitations may be related to the short follow-up duration, the study findings could help to develop evidence-based knowledge about the impact of workplace interventions using new technologies in mitigating discomfort and promoting well-being among affected workers.

**Trial Registration:**

ClinicalTrials.gov. NCT06485804 Registered on July 1, 2024.

## Introduction

### Background and rationale

Professional organizations worldwide are increasingly recognizing the importance of investing in quality assurance measures aligning closely with international standards that emphasize not only the quality of products and services [1], but also the quality of work life (QoWL) [2]. The role of workplace strategies, processes, and environment has been proven in stimulating employee job satisfaction, well-being, organizational effectiveness and overall productivity [3,4]. By adhering to stringent quality assurance directives, professional organizations strive to ensure that workplaces prioritize factors such as ergonomic design, adequate rest intervals, and access to healthcare services, all of which are essential components of promoting a healthy and sustainable work environment [5]. Work-related musculoskeletal disorders are the most common occupational disorders worldwide [6]. Neck pain (NP) is a major health problem in office workers with a one-year prevalence of 58% among data processing workers, and 33% among other office workers in the UK [7], and of 69% in Belgium [8]. Office workers have been reported to have a higher incidence of chronic pain and transition to persistent or recurrent NP over a one-year period [9]. This disorder is responsible of disability, impaired quality of life (QoL) and QoWL, with a great socioeconomic burden [10,11]. Recognizing the prevalence and the burden of this issue in modern office settings, professional organizations are implementing targeted strategies to address concerns and provide necessary support. These interventions may include ergonomic measures to optimize workstation setups, regular breaks, educational initiatives, and exercises [12].

A considerable number of studies focusing on the effects of exercise for NP in office workers have been conducted [13,14]. The evaluation of the effectiveness of various therapeutic interventions in and out the workplace, aiming to prevent or alleviate NP has been reported, including proprioceptive exercises, stretching, and dynamic-resisted strengthening exercises [15–17]. Until early June 2025, best evidence suggests that self-management for NP is recommended to include education and reassurance along with regular exercise and advice to maintain daily activities [18,19]. It has been proven that self-exercises focusing on strength and endurance training, as well as self-mobilization, were effective in reducing pain and improving function and QoL in NP patients [20]. Adherence to rehabilitation self-exercises poses a significant challenge in managing NP [21]. Patients often struggle to consistently follow prescribed exercise regimens due to factors such as lack of motivation, forgetfulness, or the perceived complexity of the exercises [22]. This non-adherence can hinder the effectiveness of rehabilitation programs and impede patients’ progress toward recovery [22]. Recognizing the need for innovative solutions, a growing interest is in incorporating new tools, such as artificial intelligence (AI) embedded smartphone application (app), into rehabilitation strategies [23]. These apps offer a promising avenue to enhance adherence by providing personalized and interactive exercise programs [24]. They represent a mode of information delivery that is suited to the information technology literate population who are familiar with and frequent users of information technology devices [25], and can be particularly useful in a working population where it may be difficult to find time to attend physiotherapy or educational sessions. While previous studies have explored the effectiveness of self-directed exercises for NP [20], some have made a direct comparison between traditional self-directed methods and smartphone-guided interventions [26]. Notably, a pilot randomized experiment comparing posture correction exercises from brochures and neck workouts from apps was carried out by Lee et al. [26]. The aforementioned study was constrained, nevertheless, by its small sample size (n = 20) and the absence of follow-up evaluations for primary and secondary outcomes, which were only assessed at baseline and just after the intervention [26].

Smartphone-guided exercises offer a potential solution by providing personalized guidance, real-time feedback, and increased motivation, which may lead to improved adherence and clinical outcomes. Thus, the use of a smartphone app to deliver a self-managed exercise and education program for office workers with chronic NP may be effective, demonstrating organizations commitment to safeguarding the health, QoL and QoWL of workers in today’s (***i.e.,*** June 2025) digitally driven workplaces.

### Objective

The objective of this study is to explore the differences in self-management using two workplace-based interventions: a smartphone app consisting of personalized neck exercises compared to conventional approach (***i.e.,*** self-exercises program on paper) in chronic NP office workers. We hypothesize that a smartphone app offering personalized neck exercises will provide superior immediate and short-term improvements in pain relief, functional ability, and QoL for office workers with chronic NP compared to conventional self-rehabilitation methods. Additionally, this approach is anticipated to address the challenge of adherence to self-exercise routines in managing this chronic condition.

### Trial design

A single blinded two-armed randomized controlled trial will be conducted. Participants will be assigned randomly to either the interventional group (IG), utilizing the smartphone app, or the control group (CG).

**Fig 1:**
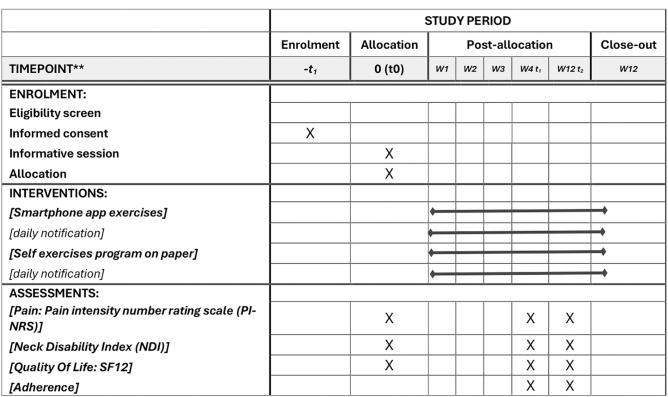
Participants timeline: schedule of enrolment, interventions, and assessments (Recommendation for interventional Trials (SPIRIT)). SF12: Short form-12. W: Week.

Fig 1 details the schedule of enrolment (Recommendation for interventional Trials).

**Fig 2:**
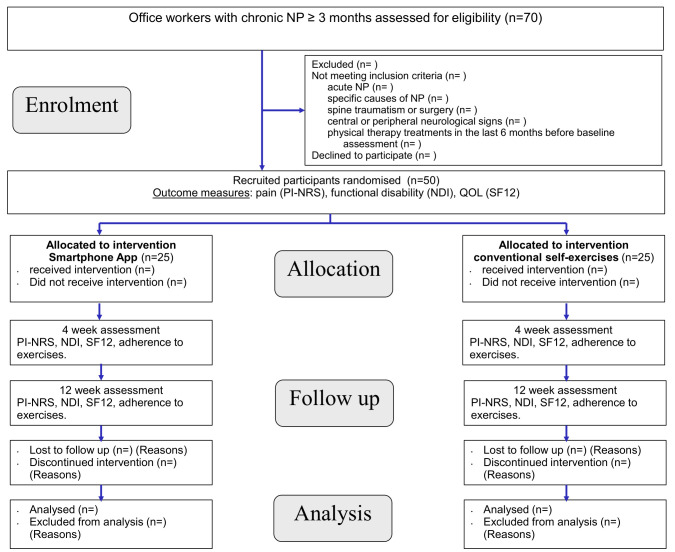
CONSORT (consolidated standards of reporting trials) follow diagram of the progress through the phases of a parallel-randomised trial of two groups. app: Application. NDI: Neck disability index. PI-NRS: Pain intensity number rating scale. QoL: Quality of life. SF12: Short form 12.

Fig 2 lists the flowchart of the study design according to “consolidated standards of reporting trials” guidelines [27].

## Methods: Participants, interventions, and outcomes

### Study setting

The study will be conducted at the University of Monastir (Tunisia). The latter is a public administration having 70 office workers, with an average (minimum-maximum) age of 45 (30–63) years, possessing an average work tenure of 15 years, engaging in eight hours of desk and computer work daily, five days a week.

### Recruitment

To recruit participants for the trial, a comprehensive digital survey will be distributed to office workers to identify individuals experiencing NP from September 1 to October 30, 2025. The survey clearly explains the trial and its objectives and expresses that all information will be analyzed with strict respect to anonymity, confidentiality, and medical secrecy. By ticking a checkbox, individuals will express their voluntary commitment to join the study, and will have access to questions identifying general information (***e.g.,*** age, sex, comorbidities, work seniority, average number of daily hours spent in front of computer); the existence of NP, duration of symptoms, pain intensity, which will be evaluated via a pain intensity number rating scale (PI-NRS), medication (***e.g.,*** pain killers, physiotherapy). Individuals identified as potentially suffering from this condition will subsequently be invited to undergo a thorough physical examination by a specialist in the field (*SS* in the authors’ list) to confirm the diagnosis.

### Eligibility criteria

Inclusion criteria will be the presence of a chronic NP lasting for more than three months, and a PI-NRS pain level at baseline assessment ≥ 3. Non-inclusion criteria will be acute NP, specific causes of NP (***e.g.,*** chronic inflammatory diseases), spine trauma or surgery, cervical radiculopathy or myelopathy, and physical therapy treatments in the last six months before baseline assessment.

### Informed consent

One researcher (*SS* in the authors’ list) will obtain written informed consents from all eligible participants before allocation. Participants will be clearly informed about the study aims and potential benefits, and that they will be free to withdraw the study at any point with no need to give any specific reason for that. They will be asked if they agree to the use of their data. This trial does not involve collecting biological specimens for storage.

### Intervention

#### Explanation for the choice of comparators.

The so-called conventional approach (self-exercises program on paper) is a standard treatment protocol for self-management in individuals with NP. The exercises are listed in a paper sheet and consist of stretching, strengthening and postural exercises.

#### Intervention description.

All participants of the IG and the CG will receive an educational session explaining the intervention details and an initial training session on how to perform exercises along with advices on proper position and ergonomics to be applied during daily work (***i.e.,*** ensure your workstation setup promotes good posture, with the monitor at eye level and the keyboard and mouse positioned close to your body; take regular breaks from sitting, standing, or staring at screens to stretch and move around). Participants will be instructed to carry out exercises at the same time daily, three to five days per week for 12 weeks.

For the CG, the rehabilitation program is delivered on a paper sheet containing pictures of exercises with a written explanation in French language with the number of sets and repetitions. Participants assigned to the IG will use the smartphone app. The latter includes the rehabilitation program with a detailed explanation in French language with the number of sets and repetitions.

Based on the findings of some papers [13,14], which focused on the effectiveness of exercise interventions for office workers with NP, we selected sets of self-exercise program with detailed explanation suitable for this population (Table 1).

**Table 1:** Neck self-exercises program.

Items	Description
***Stretching exercises***	.***Neck flexion stretch***: Sit tall in a chair; gently lower your chin towards your chest until you feel a stretch in the back of your neck. Hold for 30 seconds and repeat 3 times. .**Neck extension stretch**: Sit tall in a chair; gently tilt your head backward, looking towards the ceiling until you feel a stretch in the front of your neck. Hold for 30 seconds and repeat 3 times. .**Side neck stretch**: Sit tall in a chair; gently tilt your head towards one shoulder until you feel a stretch on the opposite side of your neck. Hold for 30 seconds and repeat on the other side. Repeat 3 times on each side
**Strengthening exercises**	.***Neck retraction exercise***: Sit tall in a chair with your back straight. Gently retract your neck by pulling your chin back without tilting your head up or down. Hold for 5 seconds and repeat 10 times. .***Isometric neck strengthener***: Place your hand against your forehead and push your head forward while resisting with your neck muscles. Hold for 10 seconds and repeat 10 times. Repeat with your hand on the back of your head (pushing backward), and on each side of your head (pushing sideways).
**Postural exercises**	.**Chin tucks**: Sit tall in a chair with your shoulders relaxed. Gently tuck your chin in towards your chest, as if making a double chin. Hold for 5 seconds and repeat 10 times. .**Shoulder blade squeeze**: Sit or stand tall with your arms by your sides. Squeeze your shoulder blades together as if trying to hold a pencil between them. Hold for 5 seconds and repeat 10 times.

### Description of the developed smartphone app

The smartphone app is designed using Android Studio and Flutter. It integrates advanced features, including an intuitive interface and an easy-to-use solution for all users (***i.e.,*** patients and healthcare professionals). Each participant in the IG will receive a code and password to log into the app and to register some general information such as age and sex. All assigned authentications will be recorded in a database created by Firebase. The app features several integrated modules:

(i) Basic information about NP and ergonomic advice on proper neck position during daily work,
(ii) Series of evaluation questions including pain assessment (PI-NRS) and the impact of NP on participant’s daily functioning,
(iii) Appropriate sets of exercises with videos and instructions,
(iv) Daily reminder feature to prompt users to engage in their prescribed exercises consistently, and
(v) Evaluation report providing feedback on user’s state and tracking their adherence.

To record participants’ engagement with the program, adherence will be monitored using a user activation tracking system assessing the frequency and duration of app use. The application is linked to the Cloud and Firebase tool enabling the therapist to visualize participants’ results in a graphical form. Exercises included are automatically adapted at each use of the app according to the participant’s condition and evolution. Indeed, the app is designed to analyze the data collected before each use from the evaluation questions based on an AI algorithm and to provide personalized exercise recommendations. To monitor and ensure participant engagement, app usage analytics will be utilized to track interaction patterns and identify any drop-offs in usage. Participants will also have access to technical support to troubleshoot any issues with the app, ensuring minimal disruption. Additionally, scheduled check-ins with participants will be conducted to address potential barriers to engagement and encourage continued adherence to the intervention protocol.

**Fig 3:**
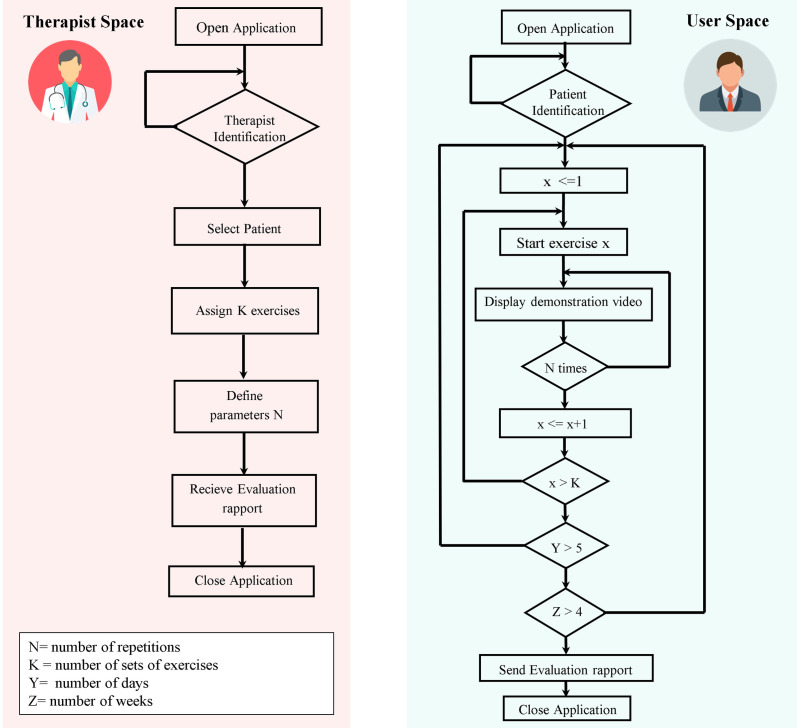
Smartphone application diagram block.

The diagram block (Fig 3) illustrates the app, which is composed of distinct therapist and participant interfaces.

### Criteria for discontinuing or modifying allocated interventions

Any requests from potential participants to discontinue participation in the intervention or follow-up will be accommodated without requiring any explanations.

### Strategies to improve adherence to interventions

Participants of both groups will receive a daily notification on their mobile phone to maximize adherence. Participants using the Smartphone app will have access to relevant information, previously described (***e.g.,*** participant evolution) to encourage them to continue with the proposed protocol.

### Relevant concomitant care permitted or prohibited during the trial

Participants will not be allowed to participate in other trials during the study period. They will be asked not to receive any extra intervention for NP during the intervention period including physiotherapy. They will be instructed to avoid using pain medication; however, if they will do, they will be asked to report the type and frequency of usage. The frequency of pain medication use before and after the intervention will be tracked to offer a more detailed understanding of pain relief and the effectiveness of the intervention.

### Outcomes

The main outcome measure is pain intensity, evaluated according to the PI-NRS [28–30]. The latter is a commonly used tool to measure a person’s pain intensity [28–30]. It is simple, quick, and widely accepted in both clinical and research settings [28–30]. Participants will be asked to indicate their current level of pain on a line from 0 (no pain) to 10 (unbearable pain) [28–30].

Secondary outcome measures are function (***i.e.,*** neck disability index (NDI)), QoL and participants’ adherence to self-exercises. The French validated version of the NDI will be used to assess the impact of NP on individual’s daily functioning [31]. The scale consists of a set of questions addressing various aspects of NP and its influence on activities such as personal care, lifting, reading, working, and recreation. Participants will be asked to rate their level of disability or limitation on a scale for each activity. The total score provides a quantitative measure of the perceived disability associated with NP, with higher scores indicating greater impairment [32]. The French validated version of the short form 12 (SF-12) will be used to measure health-related QoL. The SF-12 is a shorter version of the short form 36, which assesses an individual’s physical and mental well-being. The SF-12 is often utilized in health research, clinical practice, and population health studies [33,34]. Adherence will be measured via two methods. First, it will be evaluated using a frequency-based response scale (***i.e.,*** never, seldom, often, almost always, and always) adapted from the adherence scale of Sluijs et al. [35]. Adherence will then be treated as a dichotomous variable (***i.e.,*** adherent or not adherent), participants who will report “always” or “almost always” will be considered adherent [21]. Second, adherence will be measured based on the frequency of exercise sessions completed. Participants who engage in exercises three or more times per week will be categorized as demonstrating “full adherence” whereas those who exercise twice per week or less will be considered to exhibit “partial adherence”. These combined methods ensure a comprehensive evaluation of adherence, capturing both self-reported behavior and objective frequency of exercise.

### Participant timeline

Participants will be assessed at 4 and 12 weeks. A schematic diagram for participants’ timeline is shown in Fig 1.

### Sample size

The null hypothesis [36] was H_0_: m_1_ = m_2_, and the alternative hypothesis was H_a_: m_1_ = m_2_ + d, where “***d***” is the difference between two means and n_1_ and n_2_ are the sample sizes for the interventional and control groups, such N = n_1 _+ n_2_. The sample size was estimated using the following predictive equation [36]: $\text{n}\;=\;[\text{2}\;{\text{s}}^{\text{2}}\;{\left({\text{Z}}_{\text{1}-\alpha /\text{2}}\;+\;{\text{Z}}_{\text{1}-\beta }\right)}^{\text{2}}]/{\text{d}}^{\text{2}}$, where

- “***n***” is the number of participants;
- “***Z***_***1-α/2***_” is the normal deviate at a level of significance (type I error at 2-sided = 1.96 at 5% level of significance);
- “***Z***_***1-β***_” is the normal deviate at 1-β% power with β% of type II error (0.84 at 80% statistical power);
- “***d***” is the expected difference between average changes of PI-NRS from two groups; and
- “***s***” is the common variance (standard deviation) of PI-NRS means of two groups. “***s***” was calculated according to the following formula [36]: ${\text{s}}^{\text{2}}\;=\;[({\text{n}}_{\text{1}}-\text{1x}\;{{\text{s}}_{\text{1}}}^{\text{2}}\;+\;({\text{n}}_{\text{2}}-\text{1})\;\text{x}\;{{\text{s}}_{\text{2}}}^{\text{2}}]/({\text{n}}_{\text{1}}+{\text{n}}_{\text{2}}-\text{2})$ .

“***d***” and “***s***” values were obtained from a previous South Korean study [37] including 34 patients (17 in the IG and 17 in the CG). The patients in the IG performed exercises using a video-based app system, whereas those in the CG performed exercises using an image- and text- based printout [37]. In-home training was implemented for six weeks in both groups [37]. The NP intensity was measured before and after the intervention via the PI-NRS. Kim and Shin [37] reported that within both groups, the PI-NRS improved significantly after the intervention. At the end of the intervention, PI-NRS’ changes (expressed as means ± standard deviation) for the IG and CG were 2.23 ± 1.85 and 1.65 ± 1.26, respectively (“***d***” is therefore equal to 0.58 (0.58 = 2.23 - 1.65). Based on the aforementioned study [37], where n_1_ = 17, n_2_ = 17, s_1_ = 1.85, s_2_ = 1.26, “***s***^***2***^” is equal to 2.51 [2.51= [(17-1) x 1.85^2^ + (17-1) x 1.26^2^]/(17 + 17-2)].

The injection of the aforementioned data in the predictive equation gave a sample size of 117 participants. However, since our study involves a finite population (***i.e.,*** a population with a limited number of participant), a correction was applied to the sample size [36]. The number of participants to be included after correction is given by the formula n’ = (N x n)/(N + n), where “ ***n’*** ” represents the corrected sample size, “ ***N*** ” is the size of the finite population (which is 70 office workers in our study), and “ ***n*** ” is the sample size calculated from the standard formula (n = 117). Therefore, the corrected minimum sample size (***n’***) is equal to 44 participants [44 = (70 x 117)/(70 + 117)]. Assuming a 10% absence during the end of the intervention period, 50 participants (25 in each group) were determined to be the amended sample size [50 = 44/ (1–0.1)].

### Methods: Assignment of interventions

#### Allocation.

**Sequence generation:** The random sequence, with a 1:1 allocation of participants will be generated using a computer-generated random number in permuted blocks produced with an external website.

**Concealment mechanism:** Sealed opaque envelopes will be used to conceal the protocol order allocation.

**Implementation:** One of the coauthors (*HBS* in the authors’ list) will generate the random sequence. The principal investigator (*SS* in the authors’ list) will enroll participants and randomly assign them to one of the study groups.

#### Blinding.

One of the coauthors (*AC* in the authors’ list) who is blinded to the treatment arm will analyze data. The study principal investigator, in charge of explaining and delivering interventions (*SS* in the authors’ list), and the participants will not be blinded to the group allocation.

### Methods: Data collection, management, and analysis

#### Plans for assessment and collection of outcomes.

Evaluations will be made at baseline, at the end of the intervention period (fourth week), and at 12 weeks follow up. All evaluations will be performed digitally through a questionnaire where participants will be invited to self-report their level of pain (PI-NRS), their functional disability (NDI), their QoL (SF-12), and to rate their adherence to exercises. At the baseline education session, questionnaires and evaluation tools will be explained to participants to resolve any doubt while answering.

#### Plans to promote participant retention and complete follow-up.

Daily notifications will be intended to promote participant retention. Participants will also be given a phone number and will be informed that they can call the principal investigator if ever a doubt or a misunderstanding with their intervention protocol occurs. This availability may help participants completing the follow-up. Moreover, participants using the smartphone app will benefit from additional methods to promote self-monitoring and self-management. The information included in the app may also help to encourage participants to complete the study.

### Data management

All participants will be assigned a study identification code linked to their personal information that will be stored in a protected database accessible only to the principal investigator (*SS* in the authors’ list).

### Statistical methods

All data will be analyzed using SPSS version 23. The Shapiro-Wilk test will evaluate the normal distribution of quantitative data. If the distribution will be normal and the variances are equal, the quantitative data will be reported as mean ± standard deviation, and 95% confidence interval. Otherwise, data will be expressed as median (interquartile range). Categorical data will be expressed in percentage frequencies. The between-group differences in the mean changes of the outcome measures (PI-NRS, NDI, SF-12, adherence) after intervention will be calculated using repeated measures mixed models with participants from the IG as random effect, and group (IG or CG) and time (baseline, four weeks, and 12 weeks) as fixed effects, and with adjustments for baseline imbalance. The Spearman rank test or Pearson product-moment correlation coefficient analysis will be used to analyze the associations between clinical data and mean changes in the outcome measures. Two-sided statistical significance is set at a p value < 0.05.

### Oversight and monitoring

#### Composition of the coordinating center and trial steering committee.

The lead researcher (*SS* in the authors’ list) responsible for the study’s design will supervise the trial and manage the project as a whole. As the trial progresses, any necessary corrections will be addressed through decisions made by the entire committee (all authors).

#### Data monitoring.

**Harms:** No adverse events are anticipated, aside from potential temporary minor discomfort or fatigue. Therefore, a data monitoring committee will not be established. However, should any serious adverse events occur during the trial, the principal investigator would promptly report them to the Ethics Committee of the Faculty of Medicine of Monastir (Tunisia).

### Ethics and dissemination

#### Research ethics approval.

The study protocol has the approval of the Ethics Committee of the Faculty of Medicine of Monastir, Tunisia (IORG 0009738 N°182 OMB 0990−0279) and has been registered in ClinicalTrials.gov under the identification number NCT06485804.

#### Protocol amendments.

We do not anticipate any significant protocol modifications. However, if any changes do occur, they will need to undergo review by the Ethics Committee of the Faculty of Medicine of Monastir (Tunisia).

## Discussion

The current project will evaluate the effectiveness of a smartphone app consisting of personalized neck exercises, when compared with a conventional approach for self-rehabilitation. We expect that this innovative approach will have immediate and short-term positive effects on pain, function and QoL of office workers suffering from chronic NP. Moreover, this approach is expected to resolve the problem of adherence to self-exercises in this chronic condition.

These positive outcomes may be applicable not only to office workers with NP, but also to all NP patients. The app is a new way to treat and monitor patients’ symptoms and evolution, using new technology that facilitates constant patient-therapist communication. The integration of AI allows for dynamic adjustments based on individual progress, preferences, and real-time feedback, making the rehabilitation process more engaging and tailored to the user’s specific needs [38]. Additionally, the user-friendly interface of smartphone apps facilitates easy access to exercises, promoting greater compliance and consistency [39]. External factors such as participant motivation, technical issues with the app, and the required frequency of notifications may influence adherence to smartphone-guided exercises. To address these challenges, interventions should focus on designing user-friendly applications, providing personalized reminders, and incorporating motivational strategies such as feedback or gamification. These approaches have been suggested to improve adherence to technology-based health interventions in similar contexts [40]. Embracing these advancements in technology holds the potential to revolutionize the landscape of rehabilitation self-exercises, offering patients effective and accessible tools to actively participate in their recovery [41]. A key strength of smartphone-guided exercises is their ability to adapt to individual needs and preferences. The app’s personalized features can dynamically adjust exercise intensity, frequency, and duration based on user feedback and progress. By continuously monitoring user performance and providing real-time feedback, the app can optimize the rehabilitation program to maximize its effectiveness. This level of personalization can significantly improve adherence and motivation, leading to better outcomes in the long term [39]. Research has shown that using these tools is effective on pain, function, posture and QoL in office workers with NP [26,42] and in patients with NP in general [43]. Moreover, it has been shown that such an AI-embedded smartphone app increased the time spent on therapeutic exercise; reduced pain levels and decreased the need for other interventions in both NP and back pain [44]. Similarly, significant improvements in these conditions and in shoulder pain after using an AI-assisted health program in office workers has been reported [45]. By implementing interventions reported to have positive impact in NP within the workplace, corporations are testifying of their growing commitment to employee well-being and are anticipating benefits of cost reduction linked with absenteeism and presentism and enhancing productivity linked with a healthier workforce [46]. These interventions are gaining significance in alleviating the impact of a pathology known to occur within a work duration ≥ 5 years in office computer users independently of other risk factors [47]. The use of digital tools may also have the potential to generate positive impact on healthcare quality [48] and on healthcare systems in general by reducing direct costs (***i.e.,*** lowering face-to-face consultations, preventing unnecessary control visits), and indirect costs (***i.e.,*** patient travel, and work leaves) [49]. Findings from this study could directly inform workplace wellness policies by encouraging employers to integrate smartphone-guided exercise programs as cost-effective and accessible interventions to reduce NP among office workers. Such programs could promote regular physical activity, potentially improving productivity and reducing healthcare costs associated with musculoskeletal complaints. Clinically, these findings may support the use of digital health tools as an adjunct to standard care for NP management, aligning with evidence suggesting that technology-based interventions are effective for musculoskeletal disorders [49]. This will highlight their potential for scalable, real-world application in both occupational and healthcare settings.

### Study limitations

A notable limitation of this study could be its relatively brief follow-up period of 12 weeks. Extending the follow-up duration could offer greater insights into efficacy and participant adherence over a longer period. Subsequent studies should take this into account to comprehensively assess effectiveness and participant commitment over extended periods. Another limitation is the potential bias introduced by varying levels of participants’ familiarity with mobile apps and technology literacy, which could influence adherence rates. Future studies should consider stratifying participants by technology proficiency or providing standardized training to minimize these biases. Additionally, the assessment of the cost-effectiveness of the intervention proposed in this study will not be undertaken.

## Conclusion

In summary, by addressing the specific issue of NP associated with daily computer use, the study findings could help to get a better understanding of how to deliver self-care strategies for office workers with this chronic painful condition, and to develop evidence-based knowledge about the impact of workplace interventions using new technologies in mitigating discomfort and promoting well-being among affected workers.

## Abbreviations

AI: artificial intelligence
app: application
CG: control group
IG: interventional group
NDI: neck disability index
NP: neck pain
PI-NRS: pain intensity number rating scale
QoL: Quality of life
QoWL: quality of work life
SF12: short form 12.
